# Investigation on the Effect of Hyperbranched Polyester Grafted Graphene Oxide on the Crystallization Behaviors of *β*-Nucleated Isotactic Polypropylene

**DOI:** 10.3390/polym11121988

**Published:** 2019-12-02

**Authors:** Yansong Yu, Xi Jiang, Yiwei Fang, Jinyao Chen, Jian Kang, Ya Cao, Ming Xiang

**Affiliations:** State Key Laboratory of Polymer Materials Engineering, Polymer Research Institute of Sichuan University, Chengdu 610065, China; karlkeynes@163.com (Y.Y.); jiangxiscu2020@163.com (X.J.); spring170224@163.com (Y.F.); chenjinyao@scu.edu.cn (J.C.); caoya@scu.edu.cn (Y.C.); xiangming@scu.edu.cn (M.X.)

**Keywords:** crystallization, graphene oxide, isotactic polypropylene, hyperbranched polyester, graft

## Abstract

In this article, hyperbranched polyester grafted graphene oxide (GO) was successfully prepared. X-ray photoelectron spectroscopy (XPS), Fourier transform infrared spectroscopy (FTIR), thermogravimetric analysis (TGA), and transmission electron microscopy (TEM) were performed for its characterizations. On the other hand, differential scanning calorimetry (DSC) and wide-angle X-ray diffraction (WAXD) were also performed to study its influences on non-isothermal crystallization behaviors of *β*-nucleated isotactic polypropylene (*β*-iPP). The grafting ratios of hyperbranched polyester with different supermolecular structures were calculated to be 19.8–24.0 wt %, which increase with the degree of branching. The results showed that the grafting of hyperbranched polyester was advantageous in increasing the crystallization peak temperature *T_p_* and decreasing the crystallization activation energy *ΔE* of *β*-iPP/GO composites, which contributed to the iPP’s crystallization process. Moreover, under all cooling rates (2, 5, 10, 20, 40 °C/min), crystallinities of *β*-iPP/GO were greatly improved after being grafted with hyperbranched polyester, because of the increase of the relative contents of *α*-phase *α_c_* and the average *α*-crystal sizes.

## 1. Introduction

With the excellent properties of low cost, light weight, excellent mechanical properties, good heat resistance, and superior processability [1], isotactic polypropylene (iPP) has become one of the most widely used polymer materials [2] and has been extensively used in various areas. However, with the societal development and the increase of demand, raw iPP materials cannot meet the ever-growing demands of the production performance nowadays. For the sake of improving the iPP’s performance, there has become a new development direction in the future to compound iPP with inorganic nano-fillers [3].

Graphene is a single atomic layer consisting of sp^2^ carbon atoms, which are bonded in the hexagonal lattice [4], and it has attracted extensive attention in virtue of its extremely good thermal, mechanical, electrical, and crystallization performances [5,6,7]. It has been extensively and thoroughly studied in catalysts, polymer composites, sensors, energy devices, electronic materials, drug delivery, etc. [8,9,10,11]. It could be considered that graphene is a fine choice for reinforced fillers in polymer composites owing to its abundant material source and suitable properties.

Graphene oxide (GO) is the oxide of graphene, and it consists of oxide regions and graphite zones, with carboxyl and carbonyl on the edges as well as hydroxyl and epoxides on the matrix [12]. With the existence of these functional groups, GO becomes extremely hydrophilic and has good compatibility with polar polymers, such as polyvinyl alcohol (PVA), polyamide (PA), and polymethyl methacrylate (PMMA) [13]. Therefore, GO has been considered to be more suitable as enhancing fillers for polymers. In addition, in order to improve the dispersion of GO in nonpolar polymers, modification should be carried out by the introduction of functional groups like epoxide and carboxyl groups on the GO [5]. Surface functionalized GO (f-GO) shows outstanding interfacial compatibility in nonpolar polymers, such as polystyrene (PS) and iPP [14,15]. As a result, iPP/GO composites with great properties can be prepared by means of the addition of the f-GO into iPP [16,17,18].

Hyperbranched polyester, which has a lower branching efficiency than dendrimers, has been deeply studied due to its excellent performance and wide application [19,20]. The highly branched architecture helps to minimize low melt viscosity and chain–chain ability of the hyperbranched polyester, which is favorable for improving the compatibility with iPP [21]. The existence of multitudinous functional groups, such as hydroxyl groups, provides high reactivity with reinforced fillers, which makes hyperbranched polyester suitable as the ideal surface modifier for graphene oxide, thereby further improving the dispersion and compatibility in iPP. Therefore, it is hoped that hyperbranched polyester could be grafted onto the GO surface for modification for the sake of enhancing the iPP’s crystallization properties [22]. From previous studies, it has been found that GO is an *α*-nucleating agent of iPP, which promotes the *α*-crystallization of iPP [23,24]. It is presumed that the surface functionalization of GO by hyperbranched polyester can help to ameliorate the dispersion of GO in iPP, and improve the *α*-nucleation effect of GO, so as to further promote the iPP’s *α*-crystallization.

In summary, in this paper, the hyperbranched polyester grafted GO was prepared. We studied the crystallization behaviors of *β*-nucleated iPP/GO composites cooled under different rates (2, 5, 10, 20, and 40 °C/min) in order to investigate thoroughly the effect of hyperbranched polyester grafted GO on non-isothermal crystallization. The crystallization parameters have also been measured and calculated. 

## 2. Experimental

### 2.1. Materials

iPP, with the trade name of T38F, has an average molecular weight of 347,200 and a dispersion index of 3.63, average isotacticity of 97%, a melt index of 3.0 g per 10 min (2.16 kg, 230 °C), and was purchased from Lanzhou Petroleum Chemical Co, Ltd. (Lanzhou, China).

*β*-NA, with the trade name of WBG-II, was purchased from Guangdong Winner Functional Material Co, Ltd. (Guangdong, China). WBG-II is heteronuclear dimetal complex of lanthanum and calcium with some specific ligands, which is a kind of irregular block-like crystal whose single crystal diameter is about tens of nanometers. WBG-II has a general formula of Ca_x_La_1-x_(LIG1)_m_(LIG2)_n_, where *x* and *1*-*x* are the proportion of Ca^2+^ and La^3+^ ion in the complex, while LIG1 and LIG2 are the dicarboxylic acid and amide-type ligand with coordination numbers of *m* and *n*, respectively.

Graphene oxide (GO) was purchased from Henan Angstron Graphene Technology Co, Ltd. (Luoyang, China).

**Scheme 1:**
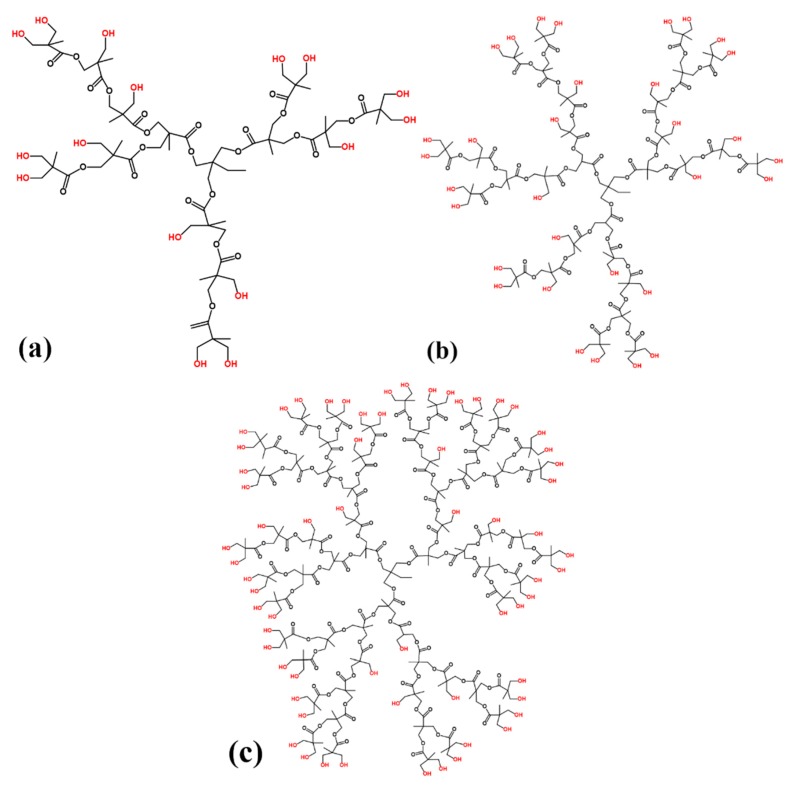
Structure diagram of hyperbranched polyester (**a**) H202, (**b**) H203, and (**c**) H204.

Hyperbranched polyester, with the trade names of Hyper H202, Hyper H203, and Hyper H204, was purchased from Shanghai Seebio Biotech, Inc., (Shanghai, China). The structure diagram of all hyperbranched polyesters is shown in Scheme 1.

*N,N*-dimethylformamide (DMF) was purchased from Sinopharm Chemical Reagent Beijing Co., Ltd. (Beijing, China).

### 2.2. Sample Preparation

**Scheme 2:**
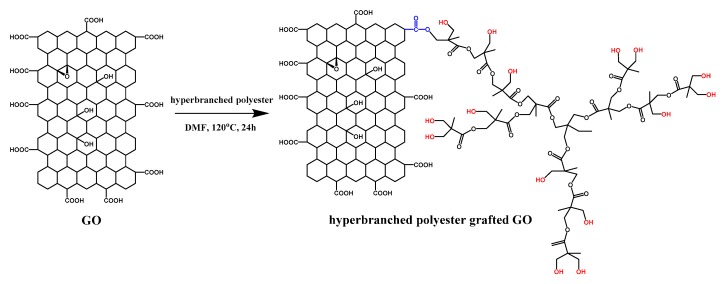
Schematic of hyperbranched polyester grafting on graphene oxide.

(1) The schematic of hyperbranched polyester grafting on graphene oxide is shown in Scheme 2. For the sake of grafting hyperbranched polyester on graphene oxide, graphene oxide (0.2 g), hyperbranched polyester (5 g), and tetrabutylammonium bromide (TBAB, 20 mg) were added in 200 mL DMF, then ultrasonically dispersed under room temperature for 1 h [25]. The obtained mixture was stirred under 120 °C for 24 h and vacuum filtered. The rinsing–filtration cycle should be repeated eight times to remove excess and physisorption of hyperbranched polyester by acetone. The resulting hyperbranched polyester grafted graphene oxide was vacuum dried under 60 °C for 12 h. Finally, hyperbranched polyester grafted GO was prepared.
(2) In order to prepare hyperbranched polyester grafted iPP/GO composites, hyperbranched polyester grafted GO, iPP, and *β*-NA WBG-II were simultaneously added into an extruder by micro-extrusion for extrusion processing at 200 °C, with 1.0 wt % GO and 0.05 wt % WBG-II. After that, the obtained sample was shaped by a pressure-molding machine. Lastly, characterization tests were carried out [26].

### 2.3. Characterization Tests

#### 2.3.1. X-ray Photoelectron Spectra (XPS)

The XPS characteristics of inorganic nano-filler GO, GO-H202, GO-H203, and GO-H204 were analyzed by the ESCALAB 250 photoelectron spectrometer (Thermo Fisher Scientific, Waltham, MA, USA) using Al K*α* as the radiation source (*hυ* = 1486.6 eV), an X-ray source of 150 W, and the pass energy of 30 eV for a high-resolution scan.

#### 2.3.2. Fourier Transform Infrared (FT-IR)

The FT-IR spectra of inorganic nano-filler GO, GO-H202, GO-H203, and GO-H204 were determined by the Nicolet iS50 FT-IR spectrometer (Thermo Scientific, Waltham, MA, USA) at a resolution of 4 cm^−1^, scanning range 4000–400 cm^−1^, and 20 scans averaged for each spectrogram [27].

#### 2.3.3. Thermogravimetric Analysis (TGA)

The TGA characteristics of inorganic nano-filler GO, GO-H202, GO-H203, and GO-H204 were measured with the TA Q5000IR thermo-analyzer instrument (TA Instruments Inc., New Castle, DE, USA) from room temperature to 800 °C at 10 °C/min under a nitrogen atmosphere (flow rate of 100 mL/min).

#### 2.3.4. Transmission Electron Microscopy (TEM)

In order to study the morphologies of inorganic nano-filler GO, GO-H202, GO-H203, and GO-H204, the transmission electron microscopy (Tecnai G2 F20, FEI, Hillsboro, OR, USA) was performed at an accelerating voltage of 120 kV. Ultrathin sections of thinner than 100 nm for TEM observation were cryogenically cut with the Leica Ultracut S ultramicrotome, then collected on 200-mesh copper grids [28].

#### 2.3.5. Differential Scanning Calorimetry (DSC)

Calorimetric experiments were all carried out on a Mettler Toledo DSC1 (Mettler, Switzerland) differential scanning calorimeter under a nitrogen atmosphere (50 mL/min). For the sake of ensuring the reliability of the data, the temperature scale calibration was carried out using indium as a standard. A 2–5 mg homogenized sample was used. The thermograms were fitted by PeakFit 4.12 software on the basis of the literature [29]. The relative contents of the *α*/*β*-phase (*α_c_*/*β_c_*) were calculated according to the following equations [30]:

(1) $$\alpha_{c}=X_{\alpha }/\left(X_{\alpha }+X_{\beta }\right)$$

(2) $$\beta_{c}=X_{\beta }/\left(X_{\alpha }+X_{\beta }\right)$$

where *X_α_* and *X_β_* represent the crystallinities of the *α*/*β*-phase, respectively.

#### 2.3.6. Wide-Angle X-ray Diffraction (WAXD)

WAXD curves were recorded with a DX-1000 diffractometer (Dandong Fangyuan Instrument Co., Ltd., China). The wavelength of CuK*α* was *λ* = 0.154 nm, and the spectrograms were recorded in the 2*θ* range 5–30°. The scanning rate was 2°/min, as well as the scanning step of 0.02°. The relative contents of the *α*/*β*-phase were calculated following standard procedures described in the literature [31]:

(3) $$\alpha_{c}=\frac{H_{\alpha }\left(110\right)+H_{\alpha }\left(040\right)+H_{\alpha }\left(130\right)}{H_{\alpha }\left(110\right)+H_{\alpha }\left(040\right)+H_{\alpha }\left(130\right)+H_{\beta }\left(110\right)}$$

(4) $$\beta_{c}=\frac{H_{\beta }\left(110\right)}{H_{\alpha }\left(110\right)+H_{\alpha }\left(040\right)+H_{\alpha }\left(130\right)+H_{\beta }\left(110\right)}$$

where *α_c_*/*β*_c_ represent the relative content of *α*/*β*-crystal form (WAXD), *H_α_*(110), *H_α_*(040), and *H_α_*(130) represent the intensities of the strongest (110), (040), and (130) diffraction peaks of the monoclinic *α*-phase, respectively. Besides, *H_β_*(110) represents the intensity of the strongest (110) diffraction peak of the trigonal *β*-phase [32].

#### 2.3.7. Non-Isothermal Crystallization Kinetics

The non-isothermal crystallization kinetics were studied in accordance with the following measures [33]:

(1) To eliminate thermal histories, all samples were heated from 50 to 200 °C under 10 °C/min and kept for 5 min.
(2) All samples were cooled down to 50 °C under 2, 5, 10, 20, and 40 °C/min, respectively, to get DSC cooling curves.
(3) All samples were heated to 200 °C under 10 °C/min to get DSC heating curves.

## 3. Results and Discussion

### 3.1. Characterizations of Hyperbranched Polyester Grafted Graphene Oxide

#### 3.1.1. XPS Survey Spectra

**Figure 1:**
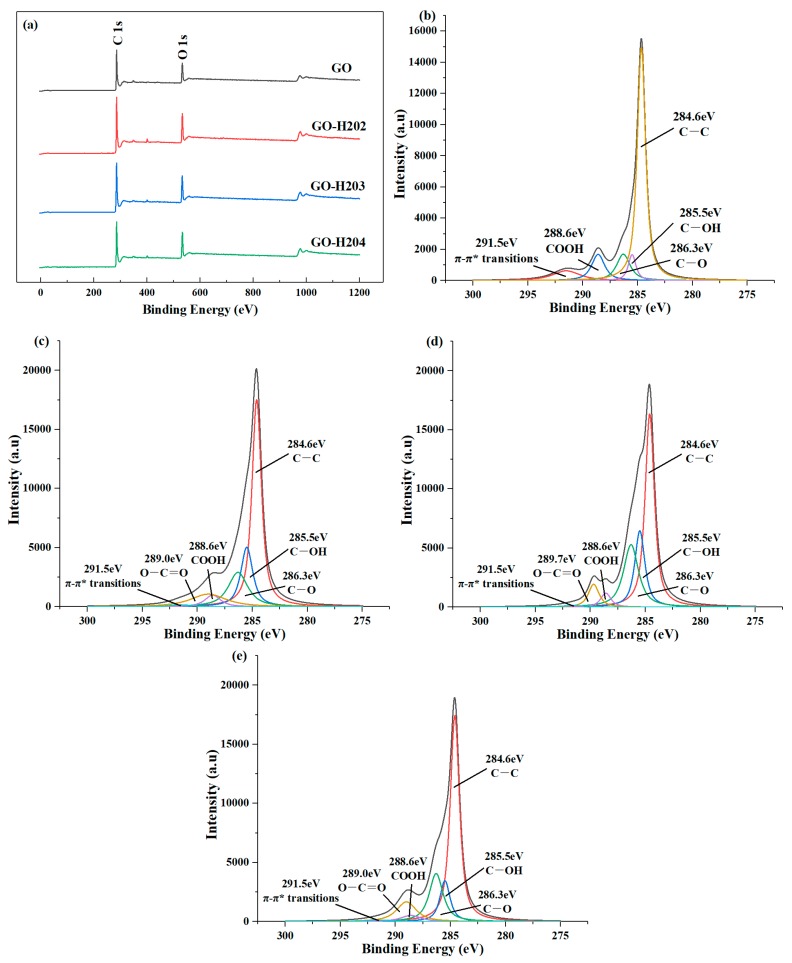
(**a**) X-ray photoelectron spectroscopy (XPS) spectrogram of various GO samples and the peak separations of C 1s, (**b**) GO, (**c**) GO-H202, (**d**) GO-H203, and (**e**) GO-H204.

The XPS spectrogram of various samples and the peak separations of C 1s for all samples are shown in Figure 1. The surface compositions and the relative atomic contents are shown in Table 1.

From Table 1, it is obvious that the intensity of the O 1s peak of the hyperbranched polyester grafted GO is substantially enhanced, which represents a significant increase in relative content of O, because of the introduction of more oxygen-containing groups to the surface of GO during the grafting process of hyperbranched polyester.

In the XPS spectrum of raw GO, as shown in Figure 1b, the C 1s peak can be fitted into five curves. The binding energies located at 284.6, 285.5, 286.3, 288.6, and 291.5 eV are corresponding to carbon skeleton (C–C), hydroxyl (C–OH), carbon–oxygen single bond (C–O), carboxyl group (COOH), and *π*-π* transition, respectively [34].

On the contrary, in the XPS spectrum of hyperbranched polyester grafted GO, as shown in Figure 1c–e, the peaks of the C–C, C–O, and *π*-π* are greatly weakened and the peak intensity of the C–OH and C–O are enhanced. More significantly, a new peak at 289.0 eV assigned to ester group (–O–C=O) appears, representing the interaction that happened between graphene oxide and hyperbranched polyester.

#### 3.1.2. FT-IR Spectra

**Figure 2:**
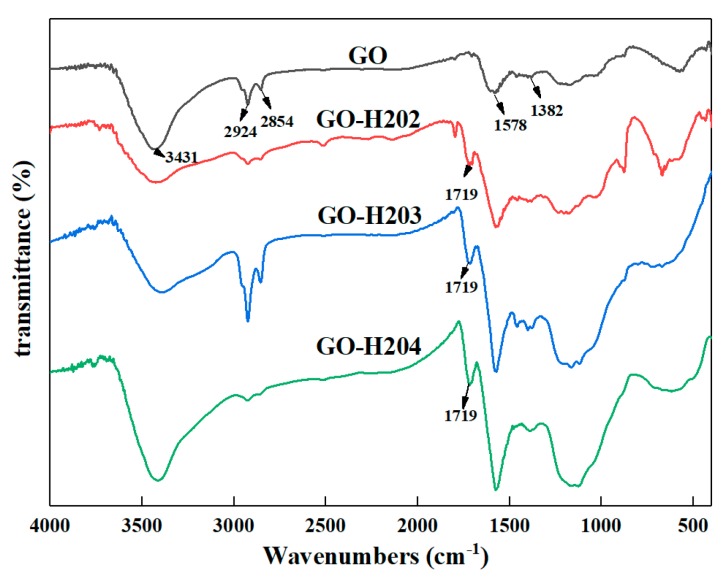
FT-IR spectrogram of various GO samples.

Figure 2 shows the FT-IR spectrogram of various GO samples. The typical peaks of GO located at 1382 and 1578 cm^−1^ could be corresponding to the C–OH stretching and C=C in the aromatic ring. The peak located at 3500–3000 cm^−1^ could be corresponding to the carboxyl. In addition, two peaks appearing at 2924 and 2854 cm^−1^ could be assigned to the –CH2 stretching.

In contrast, for hyperbranched polyester grafted GO, the peak at 1382 cm^−1^ assigned to C–OH is enhanced in the spectrum and a new peak at 1719 cm^−1^ (–O–C=O stretching vibration) appears, representing the existence of ester groups because of the interaction between hydroxyl and carboxyl.

#### 3.1.3. Thermogravimetric Analysis (TGA)

**Figure 3:**
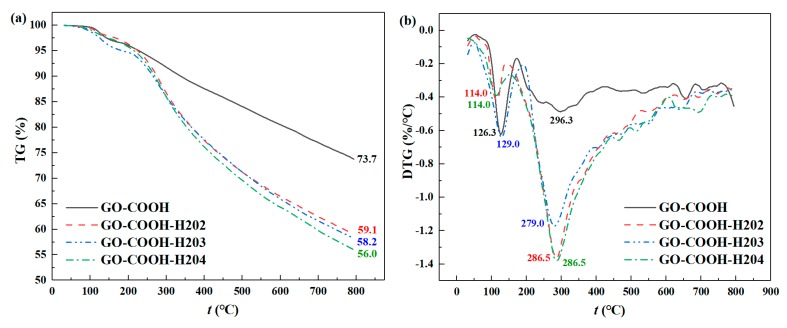
(**a**) TG and (**b**) DTG curves of various GO samples.

The thermogravimetric (TG) and differential thermogravimetric (DTG) curves of original GO and hyperbranched polyester grafted GO are shown in Figure 3. It can be seen that the thermal weight loss fraction of the original GO is 26.3% from room temperature to 800 °C, compared with over 40% thermal weight loss fraction of hyperbranched polyester grafted GO. It could be indicated that the original GO has better thermal stability. For hyperbranched polyester grafted GO, a large thermal weight loss occurs in the range 200–500 °C, reaching a maximum of -1.4%/°C at 286.5 °C, which may be caused by the high temperature decomposition of hyperbranched polyester on the surface of GO.

The grafting ratios of hyperbranched polyester could be calculated according to the weight loss rate, and the grafting ratios of H202, H203, and H204 are calculated to be 19.8, 21.0, and 24.0 wt %, respectively. It can be seen that hyperbranched polyester has been grafted on GO. Besides, the grafting ratios increase with the degree of branching.

#### 3.1.4. TEM Images

**Figure 4:**
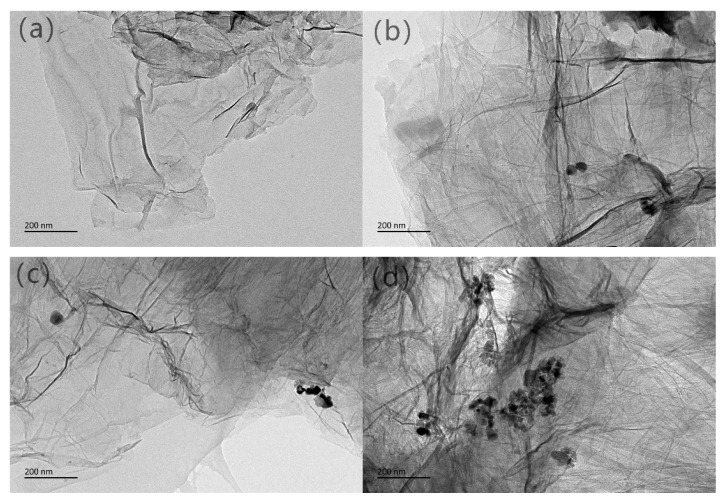
TEM images of (**a**) GO, (**b**) GO-H202, (**c**) GO-H203, and (**d**) GO-H204.

As shown in Figure 4, TEM was performed for the sake of directly observing the morphology of GO. From Figure 4b–d, it is clear that lots of dark dots can be observed compared with neat GO, which might be secondary supporting proof that the hyperbranched polyester has been grafted on graphene oxide.

### 3.2. Non-Isothermal Crystallization Kinetics

#### 3.2.1. Cooling Curves

**Figure 5:**
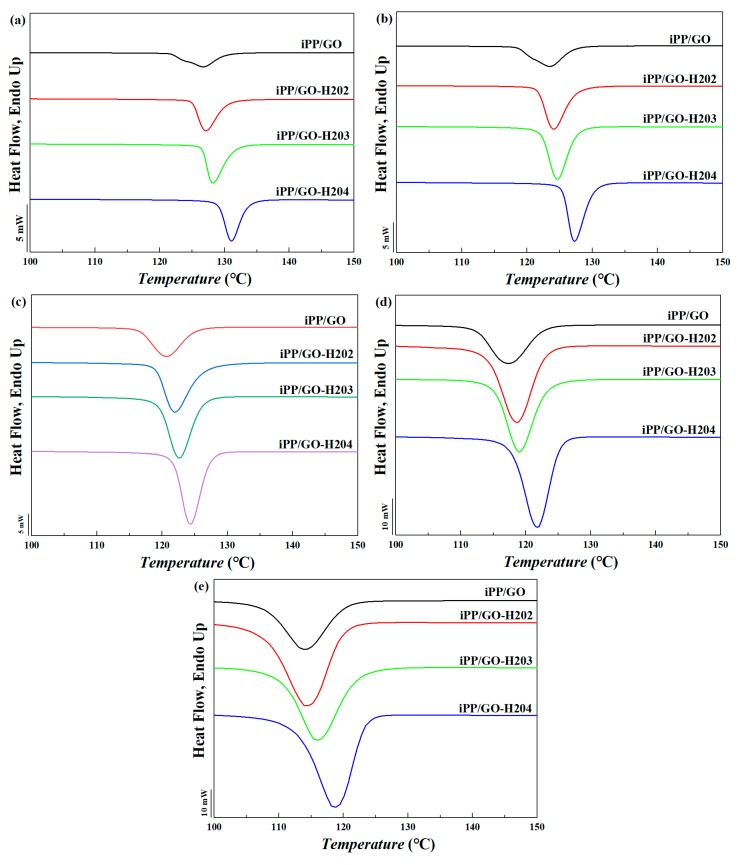
The differential scanning calorimetry (DSC) cooling curves of samples under different cooling rates of (**a**) 2 °C/min, (**b**) 5 °C/min, (**c**) 10 °C/min, (**d**) 20 °C/min, and (**e**) 40 °C/min.

**Figure 6:**
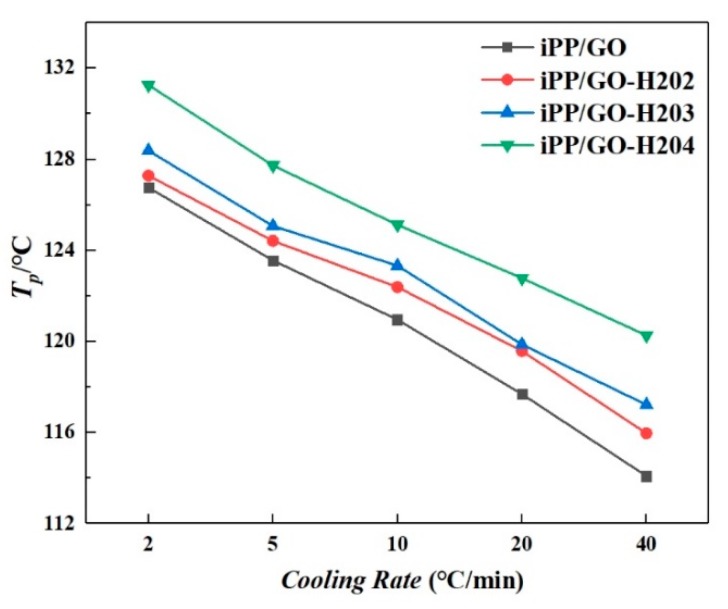
Crystallization peak temperature *T_p_* at different cooling rates as a function of cooling rate.

Figure 5 shows the cooling curves of samples cooled under different cooling rates. Figure 6 shows variations of the crystallization peak temperature *T_p_* with cooling rate.

When the cooling rate decreases, the *T_p_* of all samples increase. Meanwhile, from Figure 6, it was found that at the same rate, the *T_p_* of hyperbranched polyester grafted iPP/GO is higher than that of ungrafted iPP/GO, and the specific order is as follows: iPP/GO-H204 > iPP/GO-H203 > iPP/GO-H202 > iPP/GO. It can be concluded that the grafting of hyperbranched polyester is beneficial to increase the *T_p_* of iPP/GO composites and promote crystallization. The larger the branching degree of hyperbranched polyester, the higher the improvement effect.

The crystallization onset, peak, and endset temperatures (*T_p onset_*, *T_p,_* and *T_p endset_*) of all iPP/GO samples under different rates are shown in Table 2. With the grafting of hyperbranched polyester on GO, the *T_p onset_*, *T_p_,* and *T_p endset_* of iPP all remarkably increase, which clearly reveals the promotion of hyperbranched polyester grafting on crystallization of iPP. On the other hand, it can be seen that the width of the crystallization peak of hyperbranched polyester grafted iPP/GO is smaller than that of ungrafted iPP/GO, and the specific order is as follows: iPP/GO-H204 < iPP/GO-H203 < iPP/GO-H202 < iPP/GO. It can be inferred that the grafting of hyperbranched polyester is beneficial to enhance the lamellar thickness and improve the crystal perfection. The larger the branching degree of hyperbranched polyester, the higher the improvement effect.

#### 3.2.2. Crystallization Activation Energy

**Figure 7:**
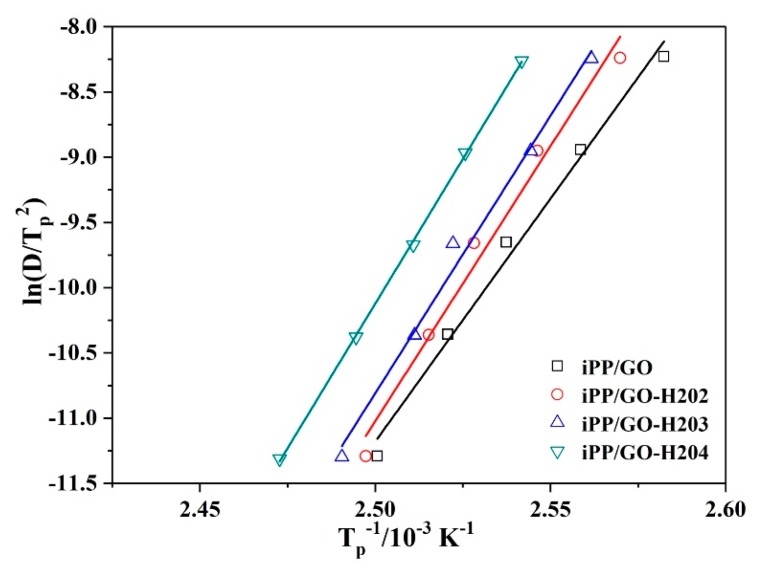
Kissinger plots for evaluating crystallization activation energies *ΔE* of all samples.

The crystallization activation energy *ΔE* represents the energy barrier to be overcome for the migration of the polymer chain from the melt to the crystal surface. The higher the *ΔE* value, the more difficult the crystallization occurs [35]. ΔE can be computed according to the Kissinger approach [36]:

(5) $$\frac{d\left[ln\left(D/T_{p}^{2}\right)\right]}{d\left(1/T_{p}\right)}=-\frac{\Delta E}{R}$$

where *D* represents the cooling rate, *T_p_* represents the crystallization peak temperature, and *R* represents the universal gas constant. Figure 7 shows Kissinger plots of ln(*D*/*T_p_*^2^) vs. *T_p_*^−1^. The ΔE can be obtained from the slope of the fitting curve. *ΔE* of all samples are shown in Table 3.

From Table 3, it is found that *ΔE* of hyperbranched polyester grafted iPP/GO is lower than that of ungrafted iPP/GO, and the specific order is as follows: iPP/GO-H204 < iPP/GO-H203 < iPP/GO-H202 < iPP/GO. It can be found that the grafting of hyperbranched polyester is beneficial to decrease the difficulty of iPP’s crystallization and to promote the crystallization. The larger the branching degree of hyperbranched polyester, the higher the improvement effect.

#### 3.2.3. Half Crystallization Time

The *X_t_* can be computed according to the following equation [37,38]:

(6) $$X_{t}\;=\;\frac{\int_{0}^{t}\left(dH/dt\right)dt}{\int_{0}^{\infty }\left(dH/dt\right)dt}$$

where *dH* represents the measured enthalpy during the crystallization time interval *dt*. The limits *t* and *∞* represent the spent time during the course of crystallization and at the end of the crystallization, respectively. Figure S1 shows variations of relative contents of crystallization *X_t_* with the crystallization time.

**Figure 8:**
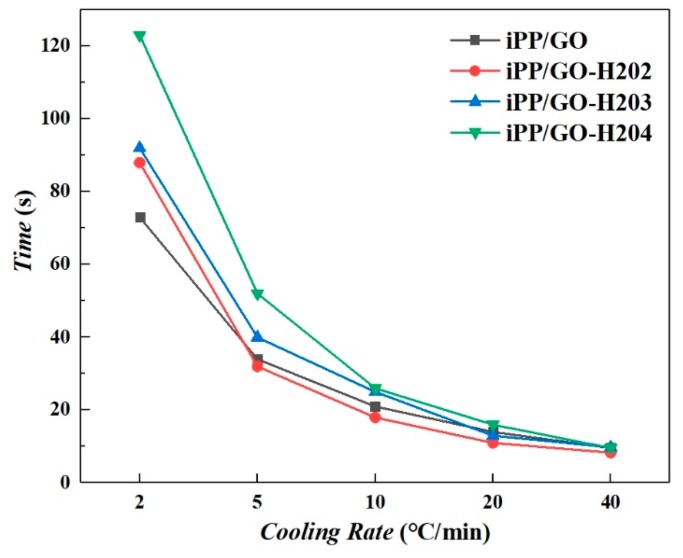
Half crystallization time *t_1/2_* as a function of cooling rate.

The half crystallization time *t_1/2_* is the time used when the *X_t_* achieves 50%. The longer the *t_1/2_*, the slower the crystallization [39]. For the sake of studying the effect of grafting of hyperbranched polyester on the crystallization rate, the *t_1/2_* of samples were calculated. Figure 8 shows variations of the *t_1/2_* with the cooling rate.

From Figure 8 it can be found that the *t_1/2_* decreases gradually with the decrease of the cooling rate. However, from Figure 8, it can be seen that at the same rate, the *t_1/2_* of hyperbranched polyester grafted iPP/GO is longer than that of ungrafted iPP/GO. This phenomenon represents the increase of crystallization time, which is contrary to the previous conclusion. It is speculated that the crystallinity and the crystallization perfection of hyperbranched polyester grafted iPP/GO are much higher than those of ungrafted iPP/GO, which leads to the prolongation of crystallization time, but not the decrease of crystallization rate.

Therefore, the crystallization parameters of the samples were tested by DSC and WAXD, and then further conclusions were drawn.

### 3.3. Crystallization Parameters

#### 3.3.1. DSC Results

**Figure 9:**
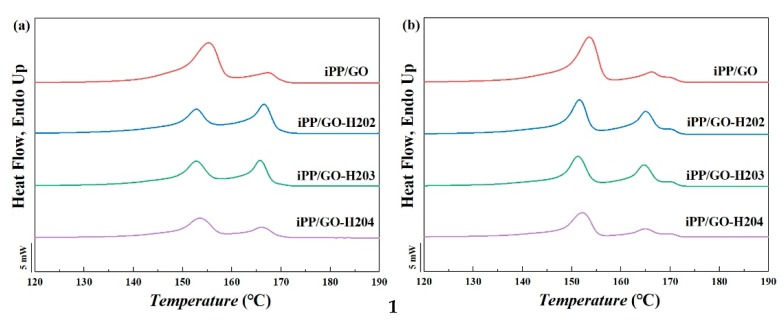

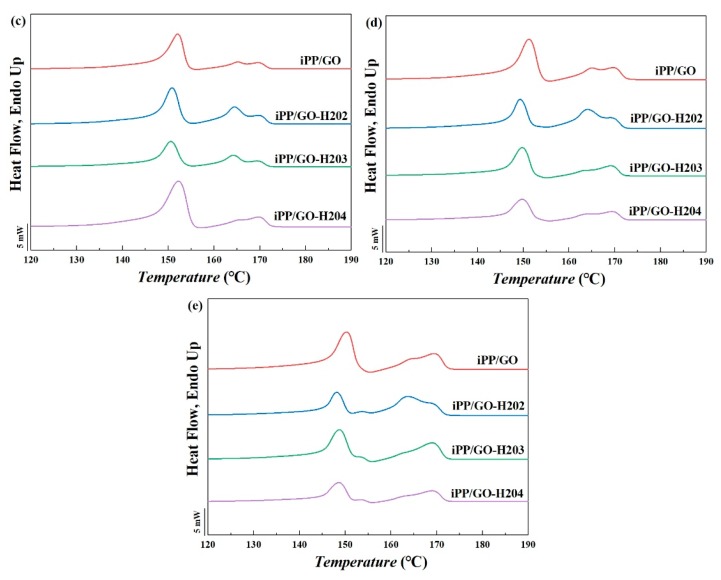
The heating curves of samples after melting and cooling under different rates of (**a**) 2 °C/min, (**b**) 5 °C/min, (**c**) 10 °C/min, (**d**) 20 °C/min, and (**e**) 40 °C/min.

**Figure 10:**
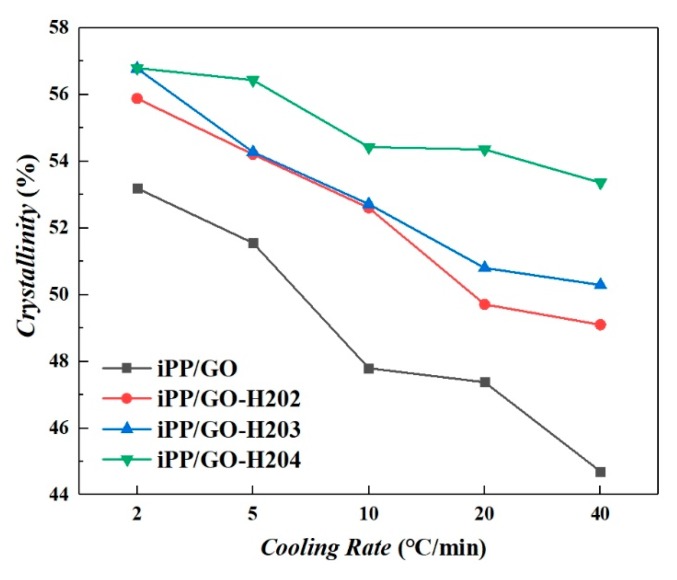
Crystallinities of samples as a function of cooling rate.

Figure 9 shows the heating curves of samples cooled under different rates. Figure 10 shows variations of crystallinities with the change of the cooling rate.

Figure 9 and Figure 10 show that as the cooling rate decreases, the crystallinity of all samples increase. Meanwhile, from Figure 10, it can be seen that at the same rate, the crystallinity of hyperbranched polyester grafted iPP/GO is much higher than that of ungrafted iPP/GO, and the specific order is as follows: iPP/GO-H204 > iPP/GO-H203 > iPP/GO-H202 > iPP/GO. It can be concluded that the grafting of hyperbranched polyester is beneficial to increase the crystallinity of iPP/GO composites and promote crystallization. The larger the branching degree of hyperbranched polyester, the higher the improvement effect.

It can be seen from Figure 9 that there are two kinds of melting peaks on the DSC curves—the peaks above 155 °C represent the *α*-phase, while those below 155 °C represent the *β*-phase [40]. Crystallinities of all samples calculated from DSC and the *α*/*β*-phase relative percentages for various GO samples cooled under different rates are shown in Table 4. It was found that crystallinities of iPP/GO were greatly improved after being grafted with hyperbranched polyester. Moreover, compared with the ungrafted iPP/GO, the relative contents of the *α*-phase of the hyperbranched polyester-grafted iPP/GO evidently increase, while the relative contents of the *β*-form decrease. The specific order of the relative contents of the *α*-phase is as follows: iPP/GO-H204 > iPP/GO-H203 > iPP/GO-H202 > iPP/GO. The larger the branching degree of hyperbranched polyester, the higher the relative contents of the *α*-phase. Considering the addition of *β*-nucleating agent in our composite system, it can be inferred that the grafting of hyperbranched polyester on GO can effectively improve the nucleation efficiency of *α*-crystallization and promote the formation of iPP *α*-crystals.

#### 3.3.2. WAXD Results

**Figure 11:**
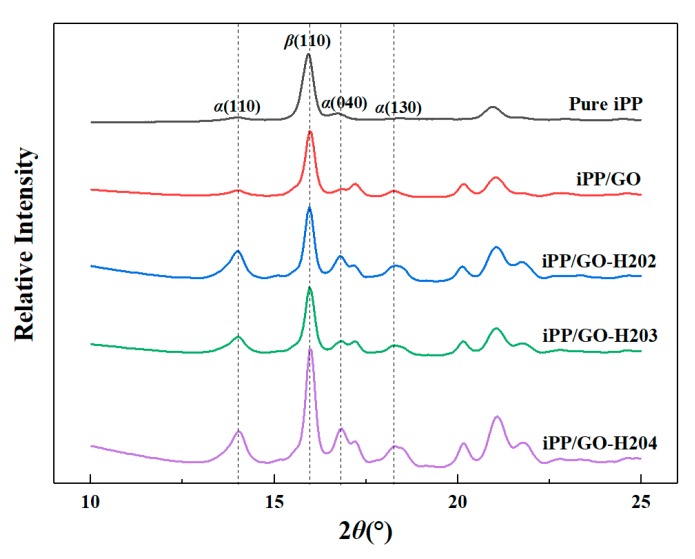
Wide-angle X-ray diffraction (WAXD) curves of all samples.

Figure 11 shows WAXD profiles of all iPP/GO samples. The relative contents of *α*/*β*-phase *α_c_*/*β_c_*, crystallinities, and average crystal sizes of samples are shown in Table 5.

It can be found that with the grafting of hyperbranched polyester, the crystallinity sharply increases, while the relative percentages of *α*-phase *α_c_* increase, as well as the relative contents of *β*-phase *β_c_* decrease, which are similar to the DSC results. Besides, the average *α*-crystal sizes of ungrafted iPP/GO are larger than that of grafted iPP/GO, but it is the opposite for the size of the *β*-phase. In summary, the grafting of hyperbranched polyester on GO contributes to the growth of iPP *α*-crystals, which leads to the promotion of the iPP’s crystallization.

#### 3.3.3. Mechanism Discussions

From the above study, the most important conclusion is that the grafting of hyperbranched polyester on GO is beneficial to the crystallization of iPP. It can be explained that GO is an α-nucleating agent of iPP. However, due to its poor compatibility with iPP, it cannot be evenly dispersed in the matrix and effectively promote the crystallization of iPP. After the grafting of hyperbranched polyester onto the surface of GO, on the one hand, the steric effect of hyperbranched polyester with a high degree of branching effectively hinders the agglomeration of GO. On the other hand, the highly branched architecture of hyperbranched polyester minimizes its chain–chain ability and low melt viscosity, which is compatible with iPP. Hyperbranched polyester can be evenly distributed on the surface of iPP matrix because of the good interface between them. Therefore, as an ideal macromolecular surface modifier, hyperbranched polyester further enhances the compatibility and dispersion of GO in iPP, thus improving the promotion of GO on the crystallization of iPP. The polarizing optical micrographs of GO samples are shown in Figure S2.

Combined with the above conclusion, it can be concluded that the hyperbranched polyester with a higher degree of branching has a higher steric effect and stronger interface effect with iPP. Moreover, there are more active groups (hydroxyl) on the edge of hyperbranched polyester with a higher degree of branching, which are more conducive to enhancing its surface interaction with GO. Therefore, the larger the degree of hyperbranched polyester grafted on GO, the better its surface modification effect on GO and the crystallization promotion effect on iPP.

In addition, because GO is an α-nucleating agent of iPP, with the improvement of the surface modification effect of hyperbranched polyester on GO, the promotion effect of GO on α-crystallization of iPP will also be improved. Therefore, the larger the degree of hyperbranched polyester grafted on GO, the higher relative contents of *α*-phase obtained.

## 4. Conclusions

In our paper, the hyperbranched polyester grafted GO was successfully prepared and characterized. Its influences on crystallization behavior and non-isothermal crystallization kinetics of *β*-iPP were also studied. The conclusions are listed as follows:

The hyperbranched polyester has been grafted on GO. The grafting rates of hyperbranched polyester H202, H203, and H204 are calculated to be 19.8, 21.0, and 24.0 wt %, respectively.

Under all cooling rates (2, 5, 10, 20, 40 °C/min), the grafting of hyperbranched polyester on GO is favorable for increasing the crystallization peak temperature *T_p_* and lamellar thickness, as well as decreasing the crystallization activation energy *ΔE* of composites, which contribute to the iPP’s crystallization process. However, the crystallization time will be prolonged after the grafting of the hyperbranched polyester, due to the higher crystallinity and the crystallization perfection of grafted-iPP/GO.

Moreover, the crystallinities of *β*-iPP/GO are greatly improved after being grafted with hyperbranched polyester. At the same time, the relative contents of *α*-phase *α_c_* and the average *α*-crystal sizes also get increased. It can be concluded that the grafting of hyperbranched polyester on GO contributes to the growth of iPP *α*-crystals, which promotes the iPP crystallization. As an ideal macromolecular surface modifier, hyperbranched polyester further enhances the compatibility and dispersion of GO in iPP, thus improving the promotion of GO on the crystallization of iPP.

## Figures and Tables

**Table 1:** Surface composition of samples determined by XPS and the relative atomic percentages for various samples.

Sample Name	O 1s (%)	C 1s (%)	C–C (%)	C–OH (%)	C–O (%)	COOH (%)	π-π^*^ (%)	O–C=O (%)
			284.6 eV	285.5 eV	286.3 eV	288.6 eV	291.5 eV	289.0 eV
**GO**	28.6	71.4	62.3	6.4	11.5	11.0	8.8	0
**GO-H202**	37.8	62.2	48.6	17.7	16.7	4.6	1.1	11.3
**GO-H203**	39.4	60.6	45.6	19.6	23.7	3.7	1.1	6.3
**GO-H204**	39.1	60.9	53.7	11.7	18.7	3.6	1.2	11.1

**Table 2:** The onset, peak, and endset crystallization temperatures (*T_p onset_*, *T_p_,* and *T_p endset_*) of all iPP/GO samples under different cooling rates.

Cooling Rate	Sample Name	*T_p onset_* (°C)	*T_p_* (°C)	*T_p endset_* (°C)	Width
**2 °C/min**	iPP/GO	130.3	126.8	121.8	8.5
	iPP/GO-H202	130.6	127.3	125.2	5.4
	iPP/GO-H203	131.6	128.4	126.8	4.8
	iPP/GO-H204	133.8	131.3	129.3	4.5
**5 °C/min**	iPP/GO	127.3	123.6	118.7	8.6
	iPP/GO-H202	127.9	124.4	121.9	6
	iPP/GO-H203	128	125.1	122.5	5.5
	iPP/GO-H204	130.6	127.7	125.8	4.8
**10 °C/min**	iPP/GO	125.4	121	116.7	8.7
	iPP/GO-H202	126.8	122.4	119.5	7.3
	iPP/GO-H203	126.9	123.3	120.2	6.7
	iPP/GO-H204	128	125.1	122.4	5.6
**20 °C/min**	iPP/GO	123.4	117.7	112.7	10.7
	iPP/GO-H202	124.1	119.6	114.9	9.2
	iPP/GO-H203	124.3	119.9	115.9	8.4
	iPP/GO-H204	126.5	122.8	118.8	7.7
**40 °C/min**	iPP/GO	120.9	114.1	107.6	13.3
	iPP/GO-H202	121.8	116	108.6	13.2
	iPP/GO-H203	123.8	117.2	110.9	12.9
	iPP/GO-H204	125.3	120.3	113.6	11.7

**Table 3:** The *ΔE* of various GO samples.

Sample	iPP/GO	iPP/GO-H202	iPP/GO-H203	iPP/GO-H204
***ΔE* (kJ/mol)**	−309.4	−350.5	−353.5	−368.4

**Table 4:** Crystallinities of all samples calculated from DSC and *α*/*β*-phase relative percentages for various iPP/GO under different cooling rates.

Cooling Rate	Sample Name	Crystallinities (%)	*α*-Phase (%)	*β*-Phase (%)
**2 °C/min**	iPP/GO	53.2	14.5	85.5
	iPP/GO-H202	55.9	28.4	71.6
	iPP/GO-H203	56.8	37.1	62.9
	iPP/GO-H204	56.8	38.6	61.4
**5 °C/min**	iPP/GO	51.6	15.5	84.5
	iPP/GO-H202	54.2	26.7	73.3
	iPP/GO-H203	54.3	33.6	66.4
	iPP/GO-H204	56.4	37.8	62.2
**10 °C/min**	iPP/GO	47.8	21.7	78.3
	iPP/GO-H202	52.6	20.8	79.2
	iPP/GO-H203	52.7	29.7	70.3
	iPP/GO-H204	54.4	30.6	69.4
**20 °C/min**	iPP/GO	47.4	26.3	73.7
	iPP/GO-H202	49.1	27.1	72.9
	iPP/GO-H203	50.3	27.9	72.1
	iPP/GO-H204	54.4	42.8	57.2
**40 °C/min**	iPP/GO	44.7	38.6	61.4
	iPP/GO-H202	49.7	39.5	60.5
	iPP/GO-H203	50.8	43.0	57.0
	iPP/GO-H204	53.4	49.0	50.9

**Table 5:** The relative contents of *α*/*β*-phase *α_c_*/*β_c_*, crystallinities, and average crystal sizes of all samples.

Sample	Crystallinity (%)	Amorphous (%)	*α_c_* (%)	*β_c_* (%)	Crystalline Sizes *L* (nm)			
					*α* (110)	*α* (040)	*α* (130)	*β* (110)
**Pure iPP**	48.8	51.2	13.2	86.8	10.8	8.2	7.3	32.9
**iPP/GO**	58.3	41.7	17.9	82.1	11.2	8.8	8.5	28
**iPP/GO-H202**	65.0	35.0	30.9	69.1	11.7	9.9	8.9	25.7
**iPP/GO-H203**	66.2	33.8	39.6	60.4	12.1	9.4	9.3	26.1
**iPP/GO-H204**	67.3	32.7	45.3	54.7	12.5	10.0	9.3	26.6

## Supplementary Materials

The following are available online at https://www.mdpi.com/2073-4360/11/12/1988/s1, Figure S1: Relative contents of crystallization Xt as a function of the crystallization time of samples cooled under different rates (a) 2 °C /min (b) 5 C /min (c) 10 °C /min (d) 20 C /min (e) 40 °C /min, Figure S2: Polarizing optical micrographs of (a) GO (b) GO-H202 (c) GO-H203 (d) GO-H204.
