# Supplementary material for: Investigation on the Effect of Hyperbranched Polyester Grafted Graphene Oxide on the Crystallization Behaviors of β-Nucleated Isotactic Polypropylene
Source: Polymers (Basel). 2019 Dec 2;11(12):1988. doi: 10.3390/polym11121988 (PMC6960721; doi:10.3390/polym11121988)
Supplement: Supplementary file 1 [file polymers-11-01988-s001.pdf]

Supporting information

Investigation on the Effect of Hyperbranched Polyester Grafted Graphene Oxide on the Crystallization Behaviors of β-nucleated Isotactic Polypropylene

Yansong Yu, Xi Jiang, Yiwei Fang, Jinyao Chen, Jian Kang*, Feng Yang, Ya Cao and Ming Xiang

State Key Laboratory of Polymer Materials Engineering, Polymer Research Institute of Sichuan University, Chengdu 610065, People's Republic of China

* Corresponding author. Email: jiankang@scu.edu.cn

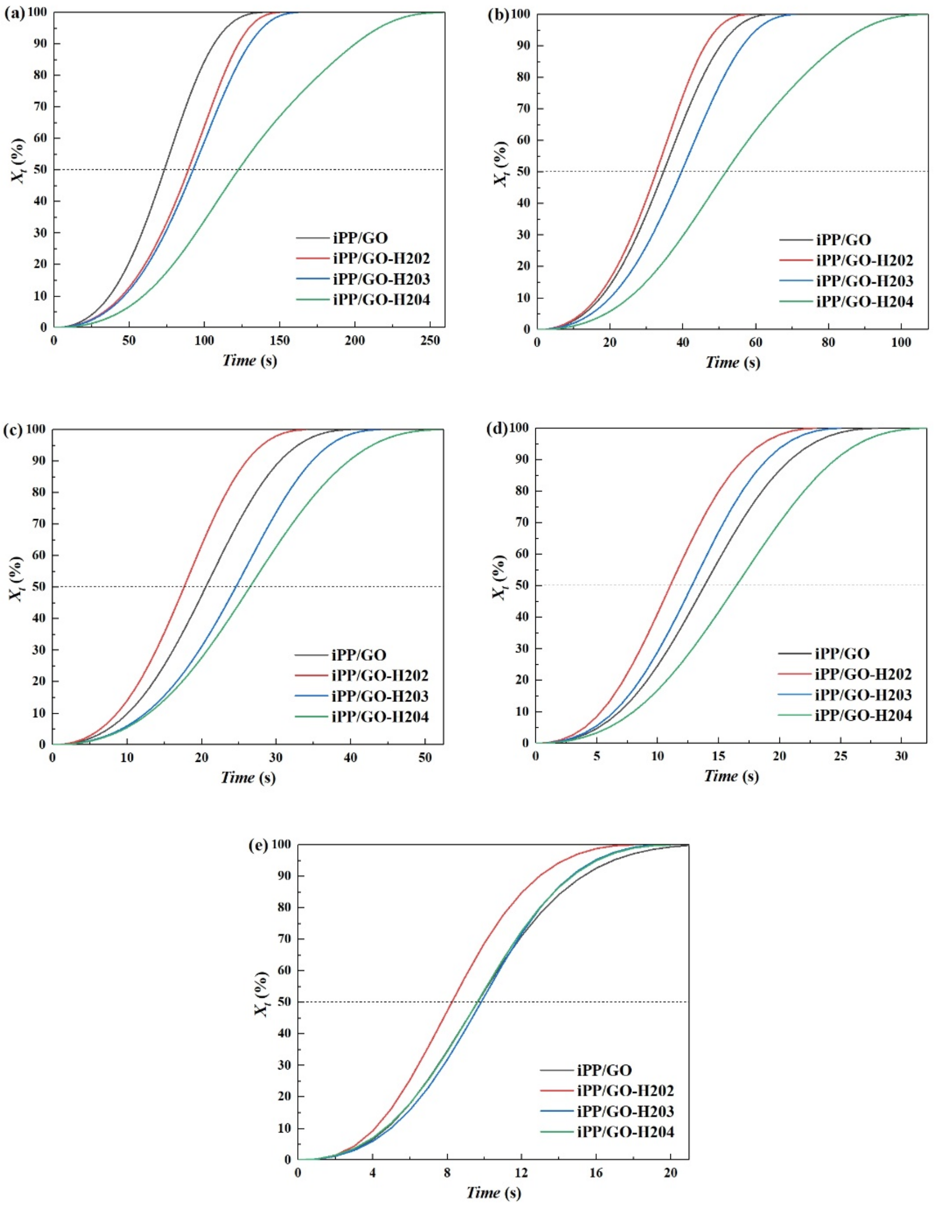

Figure S1. Relative contents of crystallization X_t as a function of the crystallization time of samples cooled under different rates (a) 2℃/min (b) 5℃/min (c) 10℃/min (d) 20℃/min (e) 40 ℃/min.

Figure S1 shows variations of relative percentage of crystallization X_t with the crystallization time.

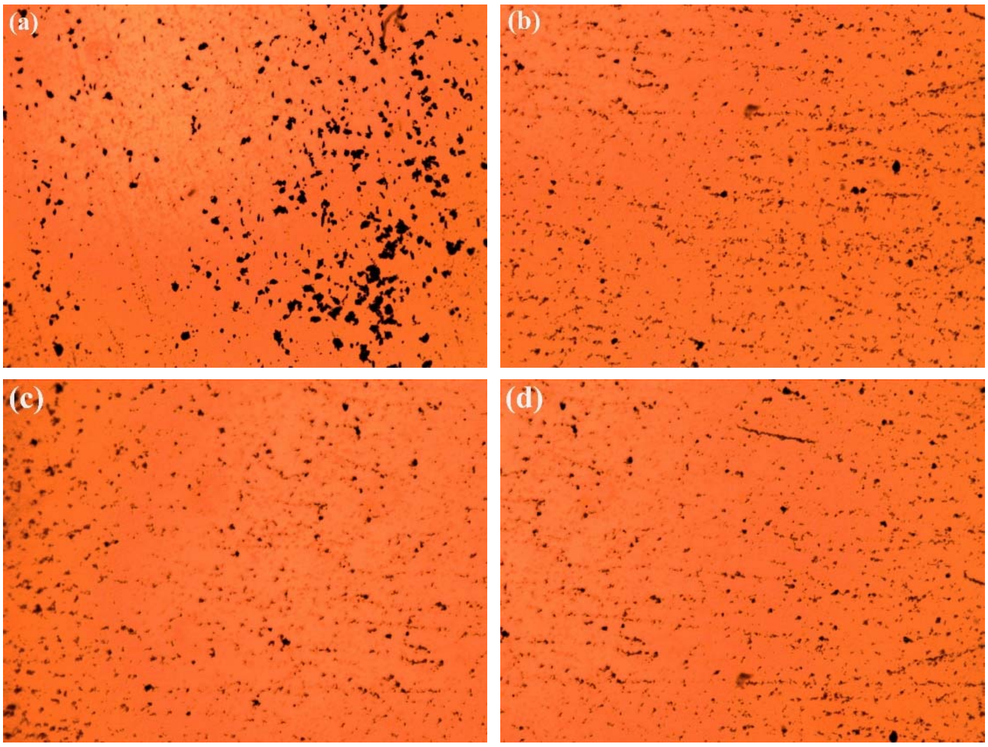

Figure S2. Polarizing optical micrographs of (a) GO (b) GO-H202 (c) GO-H203 (d) GO-H204.

The microstructure morphology of hyperbranched polyester grafted GO as well as those of neat GO sample were studied by a Olympus BX51 polarized optical microscope (POM) (Olympus Co., Tokyo, Japan) equipped with equipped with a MicroPublisher 3.3 RTV CCD. 0.1g GO and 10.0g iPP were ultrasonically dispersed in 100 mL xylene solution for 30 min, and stirred for 2 h at 130℃, then cooled to room temperature. The dispersion liquid of iPP/GO was prepared and POM was carried out to observed the dispersion of GO in iPP.

Obviously, from Figure S2(a), it can be seen that the size of black spots is large and the distribution is uneven, which indicates that the neat GO has poor dispersion in iPP and the agglomeration occurs. On the contrary, the size of black spots in Figure S2(b, c, d) a is small and the distribution is uniform, which indicates that the hyperbranched polyester grafted GO is well dispersed in iPP.
